# Natural Composition and Biosynthetic Pathways of Alkaloids in Medicinal *Dendrobium* Species

**DOI:** 10.3389/fpls.2022.850949

**Published:** 2022-05-06

**Authors:** Cheng Song, Jingbo Ma, Guohui Li, Haoyu Pan, Yanfang Zhu, Qing Jin, Yongping Cai, Bangxing Han

**Affiliations:** ^1^College of Biological and Pharmaceutical Engineering, West Anhui University, Lu’an, China; ^2^Anhui Engineering Laboratory for Conservation and Sustainable Utilization of Traditional Chinese Medicine Resources, West Anhui University, Lu’an, China; ^3^College of Life Science, Huaibei Normal University, Huaibei, China; ^4^College of Life Sciences, Anhui Agricultural University, Hefei, China

**Keywords:** alkaloid, *Dendrobium*, secondary metabolism, chemicals, pathway

## Abstract

*Dendrobium* is the second biggest genus in the Orchidaceae family, some of which have both ornamental and therapeutic values. Alkaloids are a group of active chemicals found in *Dendrobium* plants. Dendrobine has emerged specific pharmacological and therapeutic properties. Although *Dendrobium* alkaloids have been isolated and identified since the 1930s, the composition of alkaloids and their biosynthesis pathways, including metabolic intermediates, alkaloid transporters, concrete genes involved in downstream pathways, and associated gene clusters, have remained unresolved scientific issues. This paper comprehensively reviews currently identified and tentative alkaloids from the aspect of biogenic pathways or metabolic genes uncovered based on the genome annotations. The biosynthesis pathways of each class of alkaloids are highlighted. Moreover, advances of the high-throughput sequencing technologies in the discovery of *Dendrobium* alkaloid pathways have been addressed. Applications of synthetic biology in large-scale production of alkaloids are also described. This would serve as the basis for further investigation into *Dendrobium* alkaloids.

## Introduction

*Dendrobium* is the second biggest genus in the Orchidaceae family, and many of them have high medicinal and ornamental benefits (Lam et al., 2015). For almost 2,000 years, *Dendrobium* has been used as a prized traditional Chinese medicine. This can be traced back to the monograph “Shen Nong Ben Cao Jing.” The medicinal *Dendrobium* materials included in the Chinese Pharmacopoeia (11th Edition) are divided into two categories: one is the cultivated varieties from *D. nobile*, *D. huoshanense*, *D. chrysotoxum*, or *D. fimbriatum* Hook., and the fresh or dried stems of similar species in the same genus. The other is the dry stem of *D. officinale*. According to the records of the national general survey of key medicinal resources, the Ta-pieh Mountains in Anhui Province, as a natural wild progeny reserve, is home to numerous of wild *Dendrobium* species, including *D. moniliforme*, *D. officinale*, and *D. huoshanense*.

The chemical composition and categorization of different medicinal *Dendrobium* species vary massively due to the wide variety of medicinal *Dendrobium* species (Teixeira da Silva and Ng, 2017). At present, the chemical components isolated from *Dendrobium* spp. mainly included polysaccharides, alkaloids, amino acids, bibenzyls, phenanthrenes, sesquiterpenes, fluorenones, flavonoids, phenolic acids, phenylpropanoids, lignans, amides, alkaloids, steroids, etc. (Ng et al., 2012; Xu et al., 2013). Alkaloids were the first chemicals studied and structurally confirmed in *Dendrobium*. The total alkaloid content of *D. nobile* typically approached 0.5% (Wang et al., 2016). More than 40 sesquiterpene alkaloids and terpenes have been isolated and identified from *D. nobile* (Mou et al., 2021). *D. officinale* is one of the most frequently cultivated *Dendrobium* species in China. The chemical constituents have been thoroughly isolated and characterized (Wang et al., 2021). Polysaccharides, bibenzyl, phenanthrene, lignans, flavonoids, alkaloids, and other chemicals have been found in *D. officinale*. However, there have been few studies on the isolation and identification of alkaloids monomers from *D. officinale*. *D. huoshanense* is a second-class wild endangered plant in China, having extremely high medicinal and health benefits (Jin et al., 2016; Cakova et al., 2017; Yuan et al., 2018, 2019). The extraction, separation, and structural characterization of polysaccharides are the main focus of research on the active ingredients of *D. huoshanense* (Zha et al., 2017). There have also been some studies on the separation of other small molecule chemicals, such as flavonoids, terpenoids, and alkaloids (Chang et al., 2010; Song et al., 2020; Li et al., 2021). However, the biosynthesis pathway of alkaloids from *D. huoshanense* remain unclear.

Previous researches have indicated that *D. officinale* and *D. huoshanense* contain a substantial amount of nitrogen-containing compounds, including alkaloids, the composition of which varies greatly between the two *Dendrobium* species (Song et al., 2020). Aside from the complicated structure of alkaloids, the scarcity of high-quality genomes makes pinpointing key genes in the alkaloid biosynthesis pathway more challenging. Currently, the mining of alkaloid functional genes and the research of biosynthesis pathway in some model medicinal plants, such as *Artemisia annua*, *Papaver somniferum*, *Catharanthus roseus*, *Camptotheca acuminata*, and other species, has been carried out by using third-generation sequencing technologies like Pacbio and Oxford nanopore (Kellner et al., 2015a; Shen et al., 2018; Guo et al., 2018b; Kang et al., 2021). These findings have further spurred and enhanced research into the process of alkaloids production. Here, we highlighted recent progresses in the chemical identification and biosynthesis processes of *Dendrobium* alkaloids, and also discussed the alkaloid biosynthesis pathways across diverse biogenic pathways. Meanwhile, we deeply discussed the significance of high-throughput sequencing technology in uncovering the critical genes of *Dendrobium* alkaloid biosynthesis pathways. Biosynthetic gene clusters were exploited to construct the heterologous pathways for *Dendrobium* alkaloids biosynthesis based on the synthetic biology approaches, which could fulfill large-scale production and market demand.

## Composition of *Dendrobium* Alkaloids

**Figure 1:**
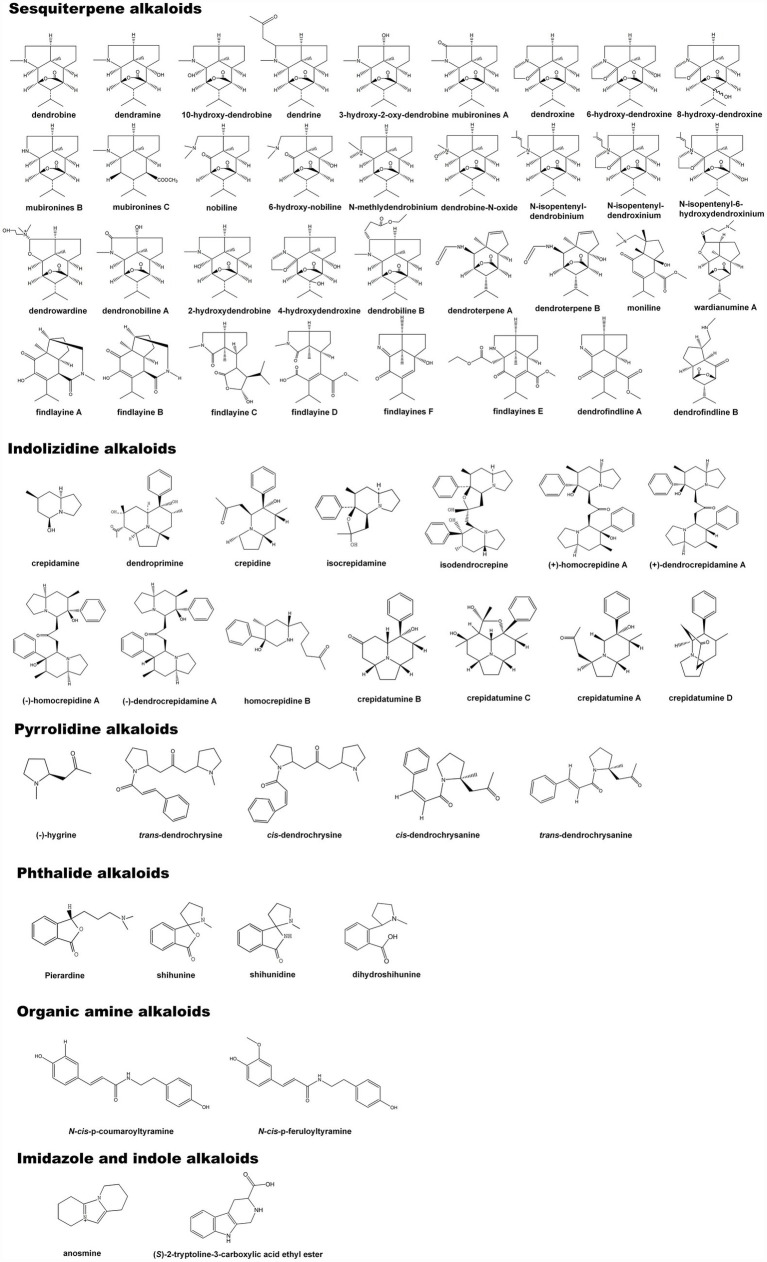
The composition and chemical structure of *Dendrobium* alkaloids.

Alkaloids are a class of nitrogen-containing alkaline organic compounds that exist in organisms (mostly plants; Facchini, 2001; Zhan et al., 2020). Most alkaloids have a complex ring structure, and nitrogen atom is mostly contained in ring. Alkaloids have a high level of biological activity and are one of the most important active substances in herbal medicines (Facchini and St-Pierre, 2005; Kiss et al., 2017). Chinese herbal medicines abundant in alkaloids have been discovered in more than 100 families with the most dicotyledonous plants, followed by monocots, gymnosperms, and ferns. Among them, *Papaveraceae*, *Leguminosae*, *Fangchiaceae*, *Ranunculaceae*, *Apocynaceae*, *Solanaceae*, and *Amaryllidaceae* are all rich in alkaloids (Kishimoto et al., 2016). Most alkaloids are derived from L-amino acids (such as tyrosine, phenylalanine, tryptophan, lysine, and arginine), or combined with steroids, secoiridoids, and other ligands (Kumar et al., 2018). Under the catalysis of a set of specific enzymes, natural amino acids can be converted into highly specialized alkaloid precursors *via* the TCA cycle (Lichman, 2021). Alkaloids are the first compounds to be isolated and validated in the structure, among which sesquiterpene alkaloids are even more studied (Kreis and Carreira, 2012). The isolation and identification of dendrobine first occurred in *D. nobile* as a significant therapeutic substance (Wang et al., 2016). Afterward, dozens of alkaloid monomers were identified from *D. parishii* Rchb.f, *D. chrysanthum* Wall, *D. crepidatum* Lind, *D. findlayanum* par.et Rchb.f, *D. friedricksianum* Lindl., *D. hilderbrandii*, and *D. loddigesii* Rolfe (Liu et al., 2007, 2020a; Zhang et al., 2017; Yang et al., 2018, 2020; Wang et al., 2019, 2020). Based on the chemical structure of the separated alkaloids, they were divided into sesquiterpene alkaloids, imidazole alkaloids, pyrrolidine alkaloids, phthalide alkaloids, indolizidine alkaloids, and other types (Mou et al., 2021; Wang et al., 2022). At least 60 alkaloids were characterized by structure, including 35 sesquiterpene alkaloids, 14 indolizidine alkaloids, five pyrrolidine alkaloids, four phthalide alkaloids, two organic amine alkaloids, one imidazole type, and one indole alkaloid (Figure 1). Among these alkaloids, the activities of total alkaloid extracts and several monomers have been verified *in vivo* or *in vitro* at various levels (Nie et al., 2018; Huang et al., 2019; Song et al., 2019; Hu et al., 2020). Some alkaloids have shown significant anti-inflammatory, anti-cancer, anti-viral, and neuroprotective properties (Table 1). Different types of alkaloids exhibit significantly different pharmacological and pharmacodynamic effects. The total alkaloids and monomers from *D. nobile*, such as dendrobine, have obvious therapeutic effects in repairing nerve and liver injuries, improving memory, suppressing LPS-induced apoptosis, and anti-virus activity (Li et al., 2011, 2017c; Nie et al., 2016; Zhou et al., 2020). In *D. crepidatum*, homocrepidines and dendrocrepidine F exhibit anti-inflammatory properties (Hu et al., 2016). Shihunine and related extracts from *D. loddigesii* have significant anti-inflammatory and diabetic symptom alleviation effects (Chen et al., 2018; Li et al., 2019). Dendrofindline A from *D. findlayanum* exhibits notable cytotoxic effects on human tumor cells while also promoting gastrointestinal motility (Liu et al., 2020a). Although several alkaloids have been identified in *D. officinale*, no relevant pharmacological effects have been reported for these substances.

**Table 1:** *Dendrobium* spp. and alkaloid constituents with biological properties.

*Dendrobium* species (organ)	Active molecule/type of extract	Experimental subjects	Activity	Ref.
*D. nobile* (stem)	Total alkaloids (90.7% dendrobine)	Rat’s cortical neurons (*in vitro*)	Attenuation of neuronal damage on cortical neurons injured by oxygen–glucose deprivation/reperfusion	Wang et al., 2010
	Total alkaloids (90.7% dendrobine)	Rat’s hippocampus	Inhibition of LPS-induced memory impairment	Li et al., 2011
	Total alkaloids (30.5% dendrobine)	Tau protein in rat’s hippocampus	Inhibition of hyperphosphorylation and LPS-induced apoptosis	Yang et al., 2014
	Total alkaloids (92.6% dendrobine)	Aβ25-35-induced memory impairment in mice	Prevention of Aβ25-35-induced neuronal and synaptic loss	Nie et al., 2016
	Total alkaloids (92.6% dendrobine)	8-week-old male Kunming mice with given doses of DNLA	Glucose-lowering and antihyperlipidemia effects in diabetic rats on the expression of the Nrf2-antioxidant pathway genes	Xu et al., 2017
	Total alkaloids (92.6% dendrobine)	Male APPswe/PS11E9 transgenic (APP/PS1) mice and wild-type (WT) littermates	Improvement of learning and memory function in APP/PS1 mice by increasing v-ATPase A1 and autolysosomal proteolysis	Nie et al., 2018
	Total alkaloids (79.8% dendrobine)	HFD-induced Male C57BL/6 mice	Balance hepatic lipid homeostasis in HFD-fed mice by increasing the taurine-conjugated bile acids and decreasing the CA/CDCA ratio	Huang et al., 2019
*D. nobile*	Dendrobine standard	Influenza A (H1N1,H3N2)	Anti-viral activity against influenza A viruses by restraining viral NP and its oligomerization	Li et al., 2017c
	Dendrobine standard	Human lung cancer cell line A549	Induced cytotoxicity and apoptosis of A549 NSCLC cells through JNK/Bim signaling pathway	Song et al., 2019
	Total alkaloids	8- to 10-week-old male wild-type and Nrf2 knockout mice	Protection of CCl4-induced mitochondrial dysfunction and mice liver injury by Nrf2 signaling pathway	Zhou et al., 2020
*D. nobile* (whole plant)	Anosmine	LPS-activated RAW264.7 cells	Inhibitory activity against NO production and a weak α-glucosidase inhibitory activity	Chen et al., 2018
*D. crepidatum* (stem)	Homocrepidine B	LPS-activated RAW264.7 cells	Moderate inhibition on LPS-induced NO production	Hu et al., 2016
	(−)-Dendrocrepidine F	LPS-activated peritoneal macrophages in mice	Higher anti-inflammatory effects by inhibiting NO production	Hu et al., 2018
	(+)-Homocrepidine A	LPS-activated RAW264.7 cells	Inhibitory effects on NO production and protective effect against LPS-induced acute lung injury in mice	Hu et al., 2016, 2020
	Isocrepidamine	HepG2 cells	Potent hypoglycemic effect *in vitro* without cytotoxicity	Xu et al., 2019
	Dendrocrepine	HepG2 cells	Potential hypoglycemic effect on high glucose model *in vitro*	Xu et al., 2020
*D. loddigesii* (stem)	Shihunine	LPS-activated RAW264.7 cells	Inhibitory activity against NO production and a weak α-glucosidase inhibitory activity	Chen et al., 2018
	Shihunine-rich extract	3 T3-L1 preadipocytes induced with insulin and 3-isobutyl-1-methylxanthine, db/db, and C57BL/6 mice	Anti-diabetic effect on db/db mice by reducing the oil droplets and TG and promoting 2-NBDG uptake in 3 T3-L1 cell	Li et al., 2019
*D. findlayanum* (whole plant)	Dendrofindline A Dendrofindline B Findlayine A	Cytotoxic effects on human tumor cell lines (A172, SHSY5Y, Hela) and zebrafish gastrointestinal motility	Inactive cytotoxicity against tumor cell lines but promoting gastrointestinal motility activities	Liu et al., 2020a
*D. Snowflake* “Red Star”	Flakinins A Flakinins B Mubironine C	Murine leukemia L1210 cells	Moderate cytotoxicity against murine leukemia	Morita et al., 2000
*D. officinale* (leaf, stem, root)	Total alkaloids	Not stated	Not stated	Shen et al., 2017
*D. officinale* (Protocorm-like bodies)	Carapanaubine, sempervirine, glycoperine, xanthoplanine, senkirkine, pelletierine	Not stated	Not stated	Jiao et al., 2018
*D. officinale* (leaf, stem)	Hordenine, piperidine, quinine, betaine Isohemiphloin, Theobromine, Trigonelline	Not stated	Not stated	Cao et al., 2019
*D. officinale* (leaf)	Total alkaloids	Not stated	Not stated	Chen et al., 2019
*D. officinale* (stem)	Caffeoylcholine 6-glucoside, cocamidopropyl βine, dopamine hydrochloride, putrescine-based derivatives	Not stated	Not stated	Zuo et al., 2020
*D. officinale* (Protocorm-like bodies)	Total alkaloids	Not stated	Not stated	Wang et al., 2021

## Sesquiterpene Alkaloids and Related Biosynthesis Pathway

**Figure 2:**
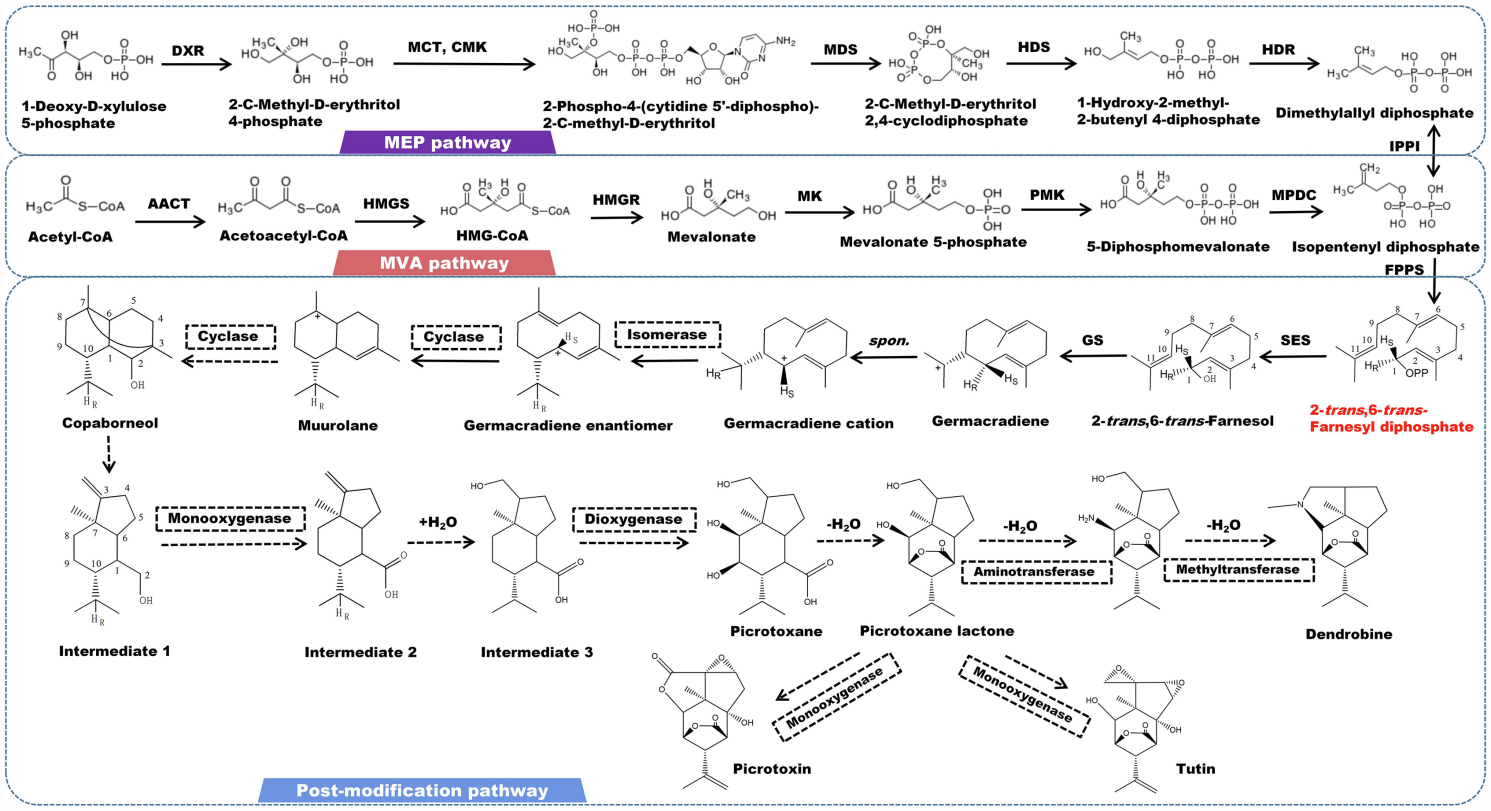
A plausible biosynthesis pathway of sesquiterpene alkaloids. The dashed box represents the putative enzymes. The dashed arrow represents the speculated pathway. Acetyl-CoA acetyltransferase (AACT), HMG-CoA synthase (HMGS), HMG-CoA reductase (HMGR), mevalonate kinase (MK), phosphomevalonate kinase (PMK), mevalonate diphosphate decarboxylase (MPDC), 1-deoxy-D-xylulose 5-phosphate reductoisomerase (DXR), 2-C-methyl-D-erythritol 4-phosphate cytidylyltransferase (MCT), 4-diphosphocytidyl-2-C-methyl-D-erythritol kinase (CMK), 2-C-methyl-D-erythritol 2,4-cyclodiphosphate synthase (MDS), 4-hydroxy-3-methylbut-2-enyl diphosphate synthase (HDS), 4-hydroxy-3-methylbut-2-enyl diphosphate reductase (HDR), isopentenyl pyrophosphate: dimethylallyl pyrophosphate isomerase (IPPI), farnesyl diphosphate synthase (FPPS), sesquiterpene synthase (SES), germacradiene synthase (GS). SES catalyzes the formation of the precursor 2-*trans*-6-*trans*-farnesol by 2-*trans*-6-*trans*-farnesyl diphosphate.

Sesquiterpene alkaloids are the most abundant alkaloids found in *Dendrobium* plants. Dendrobine is one of the most rigorously studied alkaloids, with significant pharmacological effects in anti-tumor, cardiovascular and gastrointestinal inhibitory effects, and neuroprotection (Ohc and Claisen, 2012; Song et al., 2019; Zhou et al., 2020). Picrotoxane-type sesquiterpene lactone is the fundamental skeleton of this type of alkaloid. Most alkaloids have a nitrogen atom that is bonded to the C2 and C11 positions of a sesquiterpene to create a five-membered heterocyclic ring. The nitrogen atom is commonly linked to functional groups, such as methyl and isopentenyl (Morita et al., 2000; Guo et al., 2018a; Yang et al., 2019). This conformation is considered to be directly correlated with the biogenic origins of picrotoxin and tutin (Ma et al., 2019a). The isotope tracer test demonstrated that mevalonic acid is involved in the formation of the carbon skeleton of dendrobine by using substances containing radioactive isotopes ^14^C, ^2^H, and ^3^H to cultivate and feed *D. nobile* (Meng et al., 2017). The canonical precursor of all sesquiterpene alkaloids (2-*trans*-6-*trans*-farnesol) is initially determined by 2-*trans*-6-*trans*-farnesyl diphosphate under the catalysis of farnesyl pyrophosphate synthesase (FPS). After farnesol undergo a cycloaddition event to form germacradiene, the electrons of germacrene follow the H_R_ transition from C11 to C1 to form a germacrene cation immediately. The reaction center is also shifted from C11 to C1. This stereoisomerization yields the diastereomers of the germacrene cation. Since the reaction center meets the cyclization conditions at the C1 position, the intermediate muurolane is achieved. The C2-C3 double bond is then broken, and the C3 and C7 bonds occur simultaneously, resulting in the tricyclic intermediate copaborneol. It is a common precursor in the biosynthesis of picrotoxin, tutin, and dendrobine (Wang et al., 2019). Following that, the C2-C3 bond of copaborneol is broken to form intermediate 1 (5-isopropyl-7a-methyl-1-methyleneoctahydro-1H-inden-4-yl) methanol, which is further oxidized to form intermediate 2 (5-isopropyl-7a-methyl-1-methyleneoctahydro-^1^H-indene-4-carboxylic acid), to which a H_2_O is incorporated to form intermediate 3. Dioxygenase catalyzes the hydroxylation of intermediate 3 at the C8 and C9 positions, resulting in the formation of picrotoxane (Li et al., 2017b). Picrotoxane esterifies to produce picrotoxane-lactone. Picrotoxin and tutin are synthesized by a series of oxidation reactions catalyzed by CYP450 enzymes (Figure 2). Furthermore, under the action of monooxygenase, aminotransferase, and methyltransferase, picrotoxane-lactone could ultimately be converted to dendrobine (Li et al., 2017b; Mou et al., 2021).

## Indolizidine Alkaloids and Related Biosynthesis Pathway

**Figure 3:**
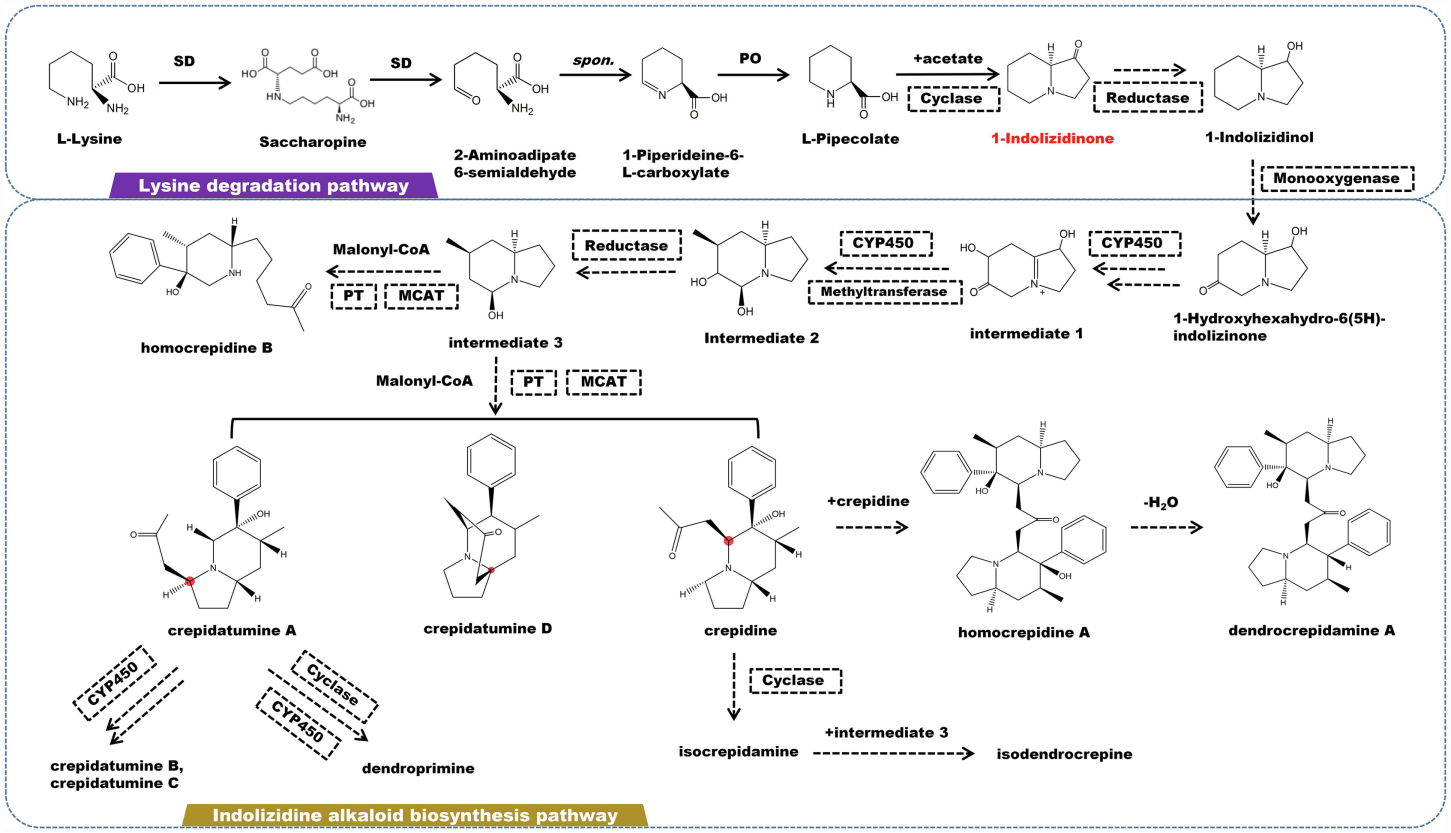
A plausible biosynthesis pathway of indolizidine alkaloids. The dashed box represents the putative enzymes. The dashed arrow represents the speculated pathway. The multiple arrows represent multi-step reactions. Saccharopine dehydrogenase (SD), L-pipecolate oxidase (PO), phenyltransferase (PT), malonyl-CoA-ACP transacylase (MCAT). Cyclase catalyzes the connection of pipecolate and acetic acid to produce the precursor 1-indolizidinone.

Indolizidine alkaloids are a type of heterocyclic compounds with specific biological and pharmacological activities that are discovered mostly in the *Apocynaceae*, *Convolvulaceae*, *Orchidaceae*, *Pandanaceae*, and *Leguminosae* (Hu et al., 2018). Currently, a large number of indolizidine alkaloids have been found in marine and terrestrial animals and plants. The fundamental structure of indolizidine alkaloids is a 5-membered ring joined with a 6-membered ring sharing a nitrogen atom (Hu et al., 2020; Zhang et al., 2021a). The biogenic pathway of indolizidine alkaloids originates from L-lysine in terms of molecular structure. L-lysine undergoes a deamination in the lysine degradation pathway, which is catalyzed by saccharopine dehydrogenase (SD) to form saccharopine. Saccharopine is catalyzed by SD to form 2-aminoadipate 6-semialdehyde, which then cyclized to form 1-piperideine-6-L-carboxylate. Under the catalysis of L-pipecolate oxidase, 1-piperideine-6-L-carboxylate degrades into pipecolate (PO). Pipecolate and acetic acid are catalyzed by cyclase to yield 1-indolizidinone. 1-Indolizidinone undergoes a multi-step reaction involving CYP450 monooxygenase, reductase, and methyltransferase to form intermediate 3 (Figure 3). It is believed that the C2 and C3 in 1-indolizidinone came from acetate *via* malonate; however, the enzyme that catalyzes this step has not been found. Intermediate 3 is catalyzed by phenyltransferase (PT) and malonyl-CoA-ACP transacylase (MCAT) to generate crepidatumine A, crepidatumine D, and crepidine, based on the location of the malonyl group binding (Xu et al., 2019, 2020). Depending on CYP450 enzyme, crepidatumine A and crepidine polymerize to form two type of enantiomeric octahydroindolizine alkaloids—homocrepidine A and dendrocrepidamine A (Hu et al., 2016). Crepidatumine A was converted to dendroprimine *via* CYP450 and cyclase (Kobayashi et al., 2012). Crepidine underwent the cyclization to yield isocrepidamine, which then combined with intermediate 3 to form isodendrocrepine (Xu et al., 2019).

## Pyrrolidine Alkaloids and Related Biosynthesis Pathway

**Figure 4:**
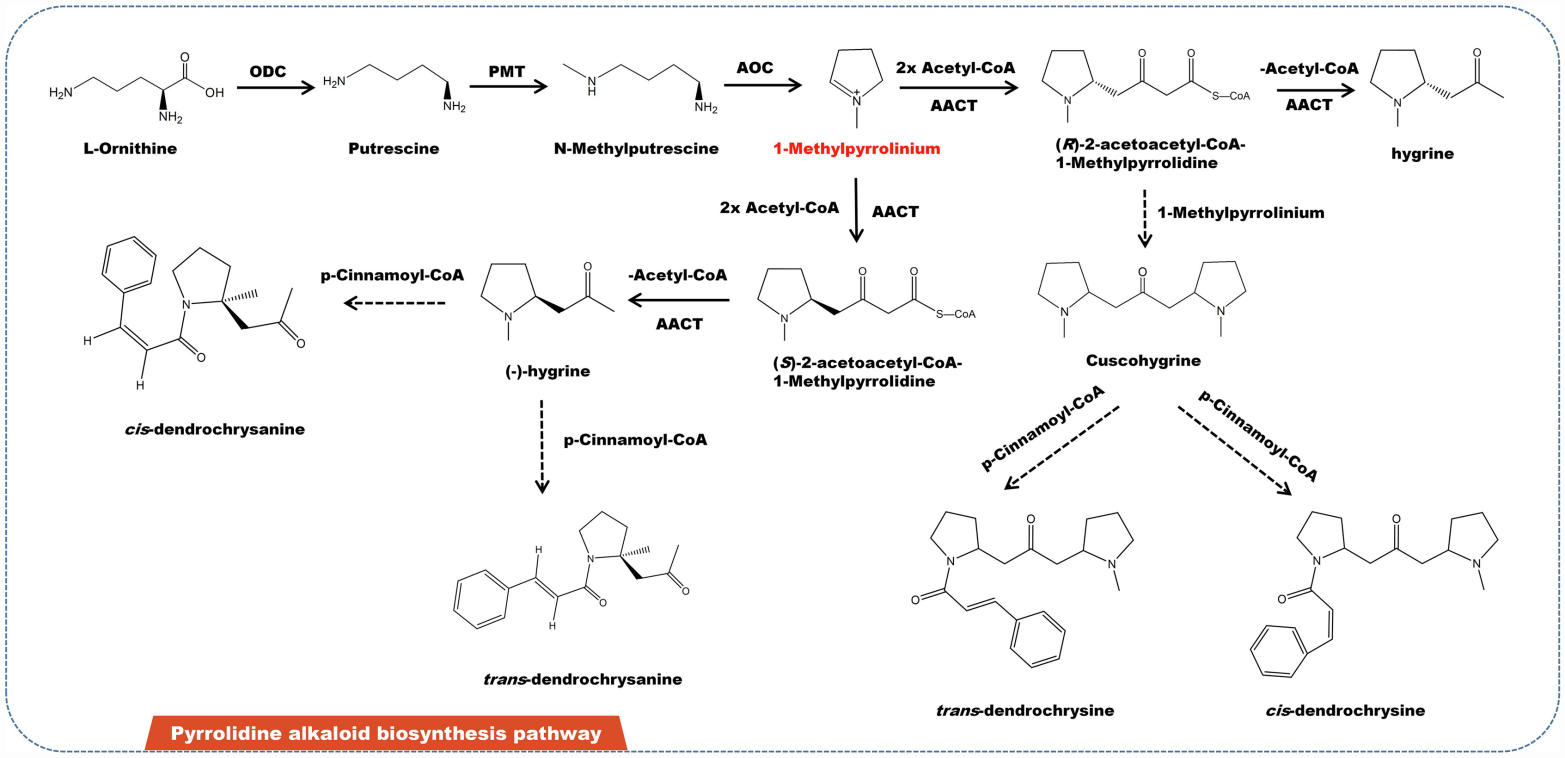
A plausible biosynthesis pathway of pyrrolidine alkaloids. The dashed box represents the putative enzymes. The dashed arrow represents the speculated pathway. Ornithine decarboxylase (ODC), putrescine N-methyltransferase (PMT), primary amine: oxygen oxidoreductase (AOC), acetyl-CoA acetyltransferase (AACT). Under the catalysis of AOC, methylputrescine is deaminated and oxidized to produce the precursor 1-methylpyrrolinium.

Pyrrolidine alkaloids are a family of structurally related substances found in *Asteraceae*, *Leguminosae*, and *Boraginaceae*. Some of them have pharmacological effects on liver toxicity to humans and livestock (Khosravi et al., 2019; Lichman, 2021). Pyrrolidine alkaloids are abundant in several edible plants, including *Senecio*, *Crotalaria*, and *Heliotropium* (Seigler, 1998). Some plants featuring pyrrolidine alkaloids are also utilized to make herbal remedies and medicinal teas. Japanese daisy tea, for example, is rich in pyrrolidine alkaloids. Currently, almost 700 pyrrolidine alkaloids have been identified from diverse plants. L-ornithine is the source of pyrrolidine alkaloids. L-ornithine is initially catalyzed by ornithine decarboxylase (ODC) to produce putrescine, which then catalyzed by putrescine N-methyltransferase (PMT) to obtain methylputrescine. Under the catalysis of primary amine: oxygen oxidoreductase (AOC), methylputrescine is deaminated and oxidized to form 1-methylpyrrolinium—the precursor of all pyrrolidine alkaloids (Figure 4). Depending on acetyl-CoA acetyltransferase (AACT), one molecule 1-methylpyrrolinium and two molecules of acetyl-CoA link to form two enantiomers (−)-hygrine and (+)-hygrine (Nguyen et al., 2015). In addition, (R)-2-acetoacetyl-CoA-1-methylpyrrolidine can also be combined with 1-methylpyrrolinium to form cuscohygrine. Cuscohygrine coupled with *p*-cinnamoyl-CoA form *trans-* and *cis-*dendrochrysine. In addition, (−)-hygrine and *p*-cinnamoyl-CoA condense to produce *cis-* and *trans-*dendrochrysanine (Yang et al., 2006).

## Biosynthesis Pathways of Imidazole and Indole Alkaloids

**Figure 5:**
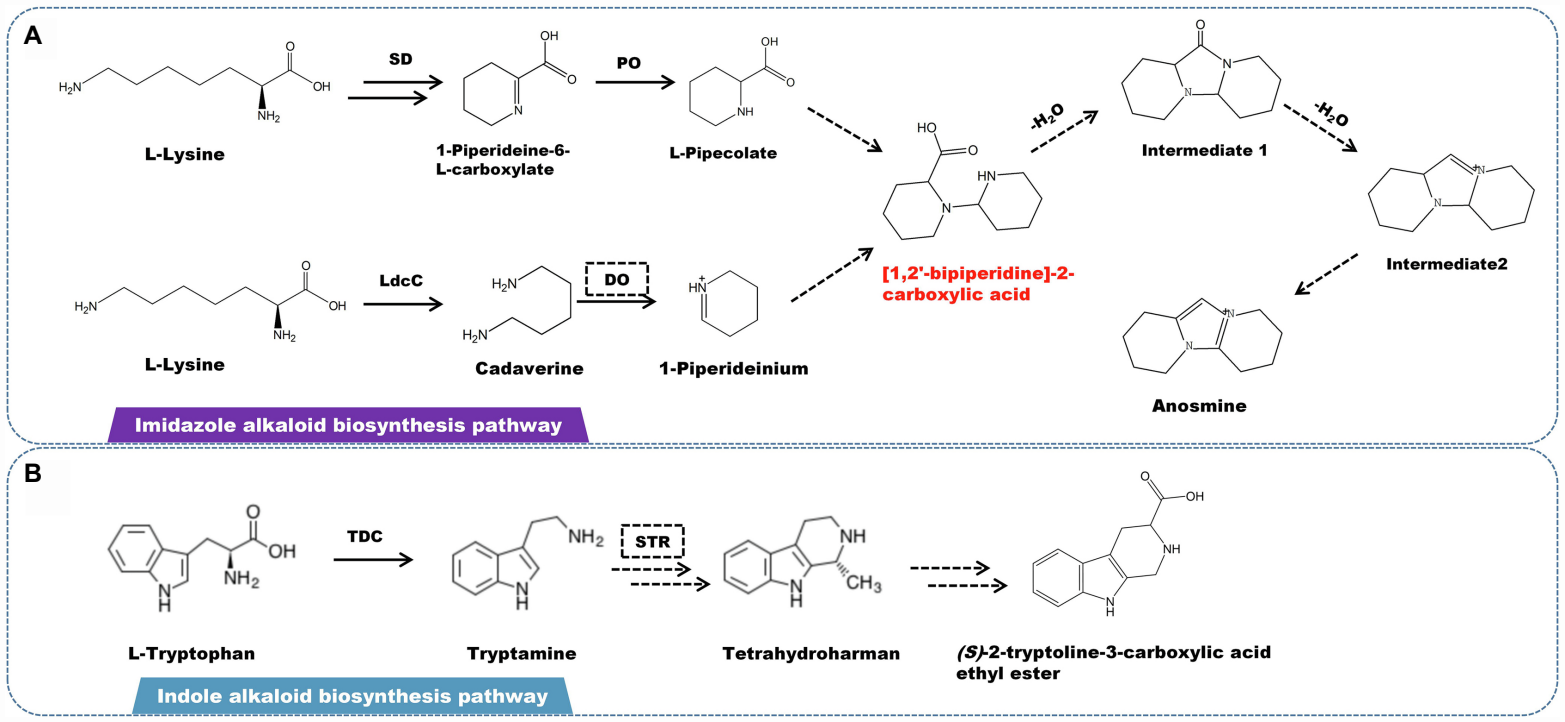
A plausible biosynthesis pathway of imidazole and indole alkaloids. **(A)** The imidazole alkaloid biosynthetic pathway. **(B)** The indole alkaloid biosynthetic pathway. The dashed box represents the putative enzymes. The dashed arrow represents the speculated pathway. The multiple arrows represent multi-step reactions. Saccharopine dehydrogenase (SD), L-pipecolate oxidase (PO), diamine oxidase (DO), lysine decarboxylase (LdcC), L-tryptophan decarboxylase (TDC), strictosidine synthase (STR). The precursor [1,2′-bipiperidine]-2-carboxylic acid is synthesized by linking L-pipecolate with 1-piperideinium derived from lysine.

A rare imidazolium-type alkaloid called anosmine was isolated from *D. nobile* (Chen et al., 2018). L-lysine is the starting point for the production of this kind of alkaloids (Hemscheidt and Spenser, 1993). Lysine decarboxylase catalyzes the conversion of L-lysine to cadaverine. Simultaneously, saccharopine dehydrogenase (SD) and L-pipecolate oxidase (PO) catalyze the formation of L-pipecolate. Cadaverine is oxidized by amine oxidase to form 1-piperideinium. L-Pipecolate and 1-piperideinium combine to form [1,2′-bipiperidine]-2-carboxylic acid, which is dehydrated to form intermediates 1 and 2. The latter is subjected to a series of oxidation to produce anosmine (Figure 5A). Other alkaloids, such as terpenoid indole alkaloids (TIAs), are also found in some *Dendrobium* spp. However, there have been few studies on the isolation and identification of indole alkaloids, with only the β-carbolin-type indole alkaloid (S)-2-tryptoline-3-carboxylic acid ethyl ester being isolated and characterized. Based on the chemical structure, the biosynthetic pathway of (S)-2-tryptoline-3-carboxylic acid ethyl ester is assumed to have originated from tryptophan metabolism (Figure 5B). L-tryptophan is converted to tryptamine *via* the tryptophan degradation pathway, which is catalyzed by L-tryptophan decarboxylase (TDC). Based on Pictet–Spengler reaction, tryptamine is catalyzed by strictosidine synthase (STR) to form strictosidine, which is then cracked to become tetrahydroharman (Pressnitz et al., 2018; Sharma et al., 2018; Yang et al., 2021; You et al., 2021). Through a multi-step enzymatic reaction, tetrahydroharman finally creates (S)-2-tryptoline-3-carboxylic acid ethyl ester.

## Phthalide Alkaloids and Related Biosynthesis Pathway

**Figure 6:**
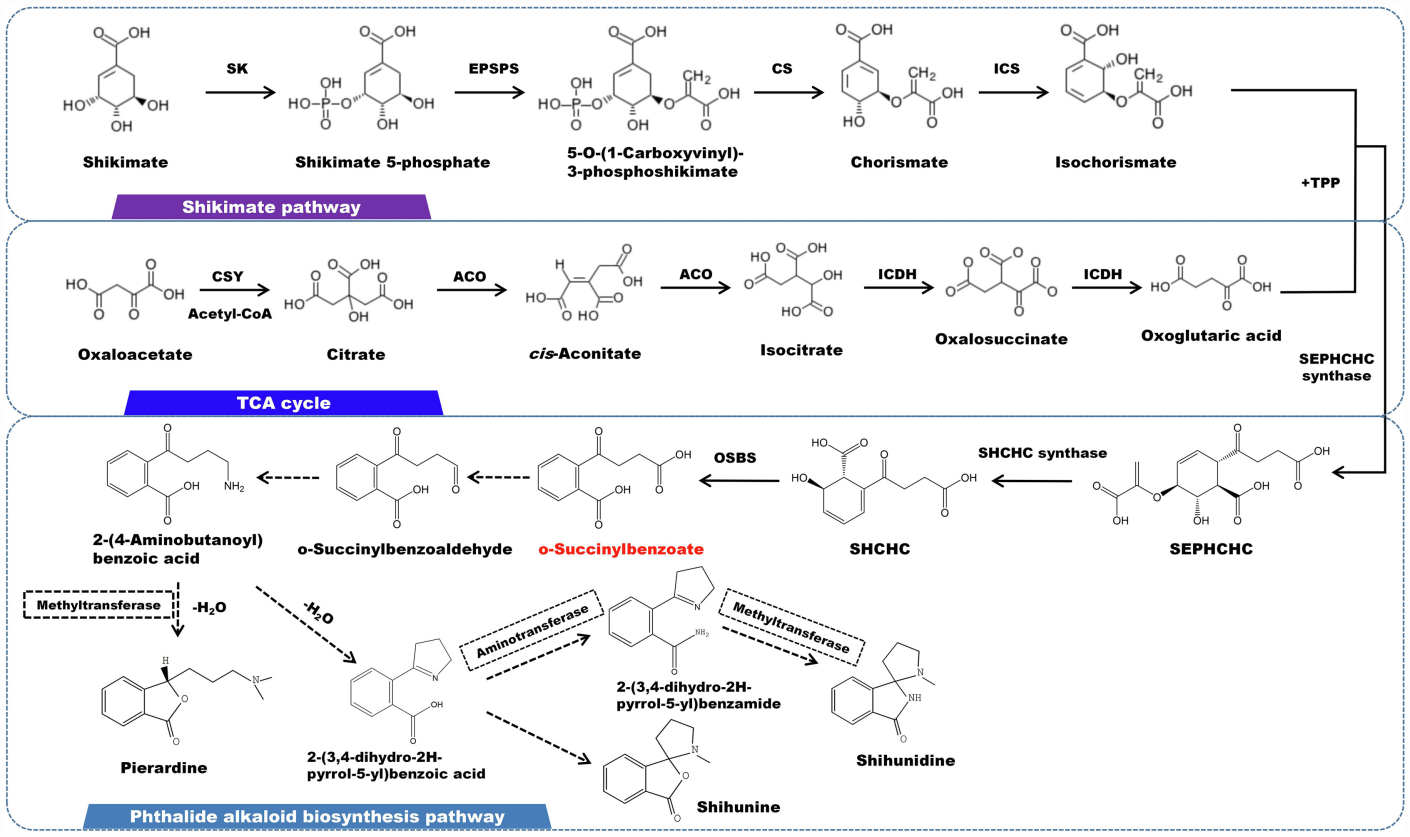
A plausible biosynthesis pathway of phthalide alkaloids. The dashed box represents the putative enzymes. The dashed arrow represents the speculated pathway. Shikimate kinase (SK), 5-enolpyruvylshikimate 3-phosphate synthase (EPSPS), chorismate synthase (CS), isochorismate synthase (ICS), citrate synthase (CSY), aconitate hydratase (ACO), isocitrate dehydrogenase (ICDH)，2-succinyl-5-enolpyruvyl-6-hydroxy-3-cyclohexene-1-carboxylic acid synthase (SEPHCHC synthase), 2-succinyl-6-hydroxy-2,4-cyclohexadiene-1-carboxylate synthase (SHCHC synthase), o-succinylbenzoate synthase (OSBS). O-succinylbenzoate served as the core structure for the biosynthesis of the phthalide alkaloid, which is produced *via* the catalysis of OSBS.

Phthalide alkaloids mainly distributed in *D. loddigesii* (Li et al., 2019). The carbon skeleton structure is o-succinylbenzoate, and the shikimic acid/o-succinyl benzoate pathway is thought to be involved in the synthesis of this kind of alkaloid (Figure 6). O-succinylbenzoate (OSB) is also the precursor of menaquinones. A number of OSB-CoA synthetase family genes have been characterized in some bacteria (Lu et al., 2012). The isochorismic acid (derived from the shikimate pathway), α-ketoglutarate (originated from TCA cycle), and thiamine diphosphate (TPP) eventually form OSB under the successive reaction of 2-succinyl-5-enolpyruvyl-6-hydroxy-3-cyclohexene-1- carboxylic acid synthase (SEPHCHC synthase), 2-succinyl-6-hydroxy-2,4- cyclohexadiene-1-carboxylate synthase (SHCHC synthase) and o-succinylbenzoate synthetase (OSBS). This process lead to the release of one molecule of CO_2_ and one molecule of phosphoenolpyruvate (PEP), respectively (Thoden et al., 2004). Following that, OSB is reduced to aldehydes and ketones, which are subsequently catalyzed by aminotransferase to generate 2-(4-aminobutanoyl) benzoic acid. The carbonyl group undergoes a reduction and combines with the oxygen on the ortho carboxyl group to produce a lactone, which is accompanied by methylation on the nitrogen atom. Pierardine is the final outcome of the chemical reaction. Simultaneously, 2-(4-Aminobutanoyl)benzoic acid can be cyclized to form 2-(3,4-dihydro-2H-pyrrol-5-yl)benzoic acid. As a precursor, 2-(3,4-dihydro-2H-pyrrol-5-yl)benzoic acid is transformed into lactone by an addition reaction, followed by methylation on the nitrogen atom to yield shihunine (Chen et al., 2018). Shihunidine is structurally related to shihunine. It is hypothesized that the hydroxyl group on 2-(3,4-dihydro-2H-pyrrol-5-yl)benzoic acid is substituted by a phenyl group, which first becomes an aniline form and then cyclizes and methylates to produce shihunidine.

## Use of High-Throughput Sequencing Technology to identify the Metabolic Pathways of *Dendrobium* Alkaloids

High-throughput sequencing has been widely employed in the discovery of novel genes and the investigation of gene structural variation in recent years (Balilashaki et al., 2019). By integrating two or more “omics,” the transcription regulators and hub genes that impact plant growth and secondary metabolism have been uncovered in many non-model plants (Kellner et al., 2015b; Saiman et al., 2018). *D. officinale*, *D. huoshanense*, *D. chrysotoxum*, and other varieties with significant medicinal benefit have undertaken *de novo* genome sequencing (Han et al., 2020; Niu et al., 2021; Zhang et al., 2021d). Combining with RNA-seq results, some researchers have systematically studied the regulatory mechanism of genes transcription and spatiotemporal characteristics under different conditions (Zheng et al., 2018; Yuan et al., 2020). The biosynthetic genes and metabolic pathways involved in *Dendrobium* alkaloids have been increasingly elucidated by the approaches described above (Zhang et al., 2021b). The genome of *D. officinale* has been upgraded in three versions, and the sequencing accuracy and assembly quality have massively improved (Yan et al., 2015; Zhang et al., 2016b; Niu et al., 2021). The *polyneuridine-aldehyde esterase* (*PNAE*) was tentatively identified based on the genomic annotation of *D. officinale* grown in Yunnan. Since *PNAE* is a crucial gene in the downstream pathway of monoterpene indole alkaloids, it is suggested that the alkaloid production of *D. officinale* may extend to the branch of 16-epivellosimine (Yan et al., 2015). Through KEGG functional enrichment on the transcriptome of *D. officinale*, 25 genes were identified as being involved in the formation of the alkaloid skeleton (Guo et al., 2013). The expression of five key enzyme genes, *geraniol 10-hydroxylase* (*G10H*), *β-subunit of tryptophan synthase* (*TSB*), *tryptophan decarboxylase* (*TDC*), *secretoganin synthase* (*SCS*), and *strictosidine synthase* (*STR*) suggested that monoterpene indole alkaloids are mainly produced in the leaves of *D. officinale*. Many studies have indicated that MeJA treatment could stimulate the expression of most genes in the MEP and MVA pathways, as well as the accumulation of total alkaloids (Jiao et al., 2018; Chen et al., 2019). A comparative transcriptome analysis of *D. officinal*e tissues and protocorm-like bodies showed 42 genes involved in the alkaloid biosynthesis pathway, and dozens of alkaloid-coding genes were identified. The expressions of some aminotransferases and CYP450 genes were significantly higher in protocorm-like bodies than in leaves, and the total alkaloid content in protocorm-like bodies likewise exhibited a similar accumulation (Wang et al., 2021). The qRT-PCR method was used to examine the expression levels of monoterpene indole alkaloid downstream pathway genes *STR*, *strictosidine-D-glucosidase* (*SG*), *PNAE*, and *vinorine synthase* (*VS*), which also revealed that the expressions of these genes are higher in protocorm-like bodies than in leaves. Transcriptome sequencing on *D. officinale* yielded a total of 2,115 unigenes related to secondary metabolism, including 4 sequences related to the indole alkaloids biosynthetic pathway, 34 sequences related to isoquinoline alkaloids biosynthetic pathway, and 38 sequences related to tropine, pyridine, and pyridine alkaloids biosynthesis (Zhang et al., 2016b). Previous studies had indicated that *DoSTR* was localized in vacuoles and that its expression was regulated by hormones, such as MeJA and ABA (Zhu et al., 2020). Recent studies generated a high-quality *D. officinale* genome at the chromosomal level, demonstrating that the sequencing data contained a contig N50 of 1.44 Mb and a Hi-C anchored rate of 93.5% (Niu et al., 2021). A total of 98 alkaloid-related genes were identified, including 56 genes encoding 25 enzymes associated with the biosynthesis of sesquiterpene alkaloids, terpenoid indole alkaloids, and their upstream pathways, including the shikimate, MVA, and MEP pathways. These genes comprise *primary amine oxidase* (*AOC*), *aspartate aminotransferase* (*AAT*), *tyrosine aminotransferase* (*TAT*), *tyrosine decarboxylase* (*TDC*), *1-benzyl-1,2,3,4-tetrahydroisoquinoline N-methyltransferase* (*CNMT*), *norbelladine O-methyltransferase* (*N4OMT*), *tropinone reductase I* (*TR1*), *PNAE*, etc. Transcriptome sequencing of *D. nobile* co-cultured with the endophytic fungus MF23 showed that a total of 16 genes were involved in the synthesis of terpenoid backbone or sesquiterpene alkaloids (Li et al., 2017b). The expression levels of *PMK* and *MVD* increased in the ninth week of co-cultivation, which was consistent with the accumulation pattern of dendrobine. The expression of *TPS21* is negatively correlated with the biosynthesis of dendrobine, and the expression of *AACT* is positively correlated with the biosynthesis of dendrobine. Further analysis of 9 post-modification enzymes found that the expression levels of *CYP1D10*, *METTL23*, *ATX4*, and *BCAT2* were higher than other culture stages after 9 weeks inoculation. The expression of *ATX4* was at a low level at 6 weeks and was activated at 9 weeks. It demonstrates that *ATX4* is essential in the modulation of the biosynthesis of *Dendrobium* alkaloids that is altered by MF23. Besides the crucial genes, some transcription factors have important regulatory roles in stress responses and secondary metabolite production in *Dendrobium* plants (Zhang et al., 2021d). DcTCP4 and DcTCP9 are involved in JA-dependent leaf developmental processes (Zhang et al., 2021b). In *D. catenatum*, the bHLH-MYB-WD40 module is deeply engaged in anthocyanin biosynthesis. DcTT8, a IIIf bHLH transcription factor, strongly regulates the expression of the anthocyanin-related genes *F3’H* and *UFGT* (Jia et al., 2021). By binding to the promoters of *CHS*, *CHI*, and *F3H*, a light-induced WD40-repeat TF DoTTG1 regulates their expression and promotes anthocyanin formation (Jia et al., 2021). MYB2 could interact with bHLH1 to enhance the expressions of *DFR* and *ANS* (Li et al., 2017a). WRKY TFs are well-studied transcriptional regulators that are mainly involved in the stress of *D. officinale* and the production of active components. Some WRKY genes commonly activated by cold stress and MeJA treatment, while others may be involved in polysaccharide biosynthesis and hydrolysis (He et al., 2017; Wang et al., 2018). DoMYB25 participated in the positive regulation of water-soluble polysaccharides (He et al., 2019). DcWRKY22, DcWRKY36, and DcWRKY45 are involved in plant drought, cold, and salt stress (Zhang et al., 2022). DobHLHs are induced by ABA and MeJA treatments. DobHLH4 positively regulates the expression of *TPS10*, therefore boosting linalool production (Wang and Liu, 2020; Yu et al., 2021). So far, there are limited reports on transcription factors that regulate dendrobium alkaloids biosynthesis. We previously isolated a MYC2 transcription factor from *D. officinale*. Overexpression of *DoMYC2* in *A. thaliana* reduces the expression of *HMGR2*, *FPS1*, and *FPS2*, implying that DoMYC2 functions as a negative regulator to regulate the expression of JA-responsive genes. DoMYC2 typically binds to the E-box in the promoters to influence transcription (Zhu et al., 2017). We highlight the classification of alkaloids and main enzyme coding genes of different *Dendrobium* species found by high-throughput sequencing in recent years (Table 2).

**Table 2:** Identification of alkaloid-related compounds and key genes in *Dendrobium* spp.

Species	Sequencing platform or technique	Classification	Annotated genes	Ref.
*D. nobile*	Molecular Cloning	Pyrrolidine alkaloid	*TR1*	Chen et al., 2013
*D. nobile*	Molecular Cloning	Pyrrolidine alkaloid	*TR2*	Cheng et al., 2013
*D. officinale*	Roche 454 GS FLX Titanium	terpenoid indole alkaloids	*AACT,HMGS,HMGR,MVK,PMK, MVD,IPI,DXS,DXR,CMS,CMK, MCS,HDS,FPS,TSB,TDC,G10H, SCS,STR,10-HGO*	Guo et al., 2013
*D. officinale*	Illumina Hiseq 2000 and PacBio Sequel	Monoterpene indole alkaloids	*AACT,HMGS,HMGR, MVK, PMK, MVD,IPI, DXS,DXR,CMS,CMK, MCS,HDS,FPS, PNAE*	Yan et al., 2015
*D. officinale*	Molecular Cloning	Precursor of terpenoid	*DXS,DXR*	Fan et al., 2016
*D. catenatum*	Illumina HiSeq 2000	Precursor of terpenoid	*TPS-a, TPS-b, TPS-e/f, TPS-c, TPS-g*	Zhang et al., 2016a
*D. officinale*	Illumina Hiseq 2000	Indole alkaloids, isoquinoline alkaloid, tropane, piperidine, and pyridine alkaloid	*Not stated*	Zhang et al., 2016b
*D. nobile*	Agilent Bioanalyzer 2,100	Sesquiterpene alkaloids	*AACT,HMGS,HMGR,MK,PMK, MVD,GPPS,FPS,TPS21,CYP71D55,CYP735A,CYP71D10,CYP94C1, METTL23,ATX4,AAT2,DAT, BCAT2*	Li et al., 2017b
*D. officinale*	Agilent Bioanalyzer 2,100	terpenoid indole alkaloids	*AACT,HMGS,HMGR,MVK,PMK, MVD,IPI,DXS,DXR,CMS,CMK, MCS,HDS,FPS,TSB,TDC,STR*	Shen et al., 2017
*D. officinale*	Molecular Cloning	Precursor of terpenoid	*MYC2*	Zhu et al., 2017
*D. huoshanense*	Illumina Hiseq 2,500	terpenoid indole alkaloids	*FPS,AACT,HMGS,HMGR,MVK, PMK,MVD,IPI,DXS,DXR,CMS, CMK,MCS,TDS,TSB,HDR*	Yuan et al., 2018
*D. officinale*	Illumina Hiseq 4,000	Sesquiterpene alkaloids	*AACT,HMGS,HMGR,MVK,PMK, MVD,DXS,DXR,MCT,CMK,MDS, HDS,HDR,FPS,IPI*	Chen et al., 2019
*D. officinale*	Molecular Cloning	Monoterpene indole alkaloids	*STR*	Zhu et al., 2020
*D. officinale*	Genome-wide identification	Precursor of indole alkaloids	*GAD,HDC,TYDC,TDC*	Zhang et al., 2020
*D. officinale*	Genome-wide identification	Precursor of terpenoid	*TPS-a, TPS-b, TPS-c, TPS-e/f*	Yu et al., 2020
*D. chrysotoxum*	MGISEQ-2000 and PacBio Sequel	Precursor of terpenoid	*TPS-a, TPS-b, TPS-c, TPS-e/f, TPS-g*	Zhang et al., 2021c
*D. officinale*	Illumina Hiseq 4,000 and PacBio Sequel II	Sesquiterpene alkaloids, isoquinoline alkaloid, pyrrolidine alkaloid, terpenoid indole alkaloids	*AOC, AAT, TAT, TDC, CNMT, N4OMT,TR1,PNAE,HMGS,HMGR,MVK,PMK,MVD,DXS,DXR, CMS,CMK,MCS,PNAE,SDR,AAE, TPS21,FDFT1,SQLE,FLDH, CYP71D55*	Niu et al., 2021
*D. officinale*	BGIseq 500	Monoterpene indole alkaloids	*TS,TDC,STR,HDR,GPPS,GES, G10H,UGT8,7-DLH,LAMT,SG, GS,PNAE,VS,AACT,HMGS, HMGR, MVK,PMK,MVD,IPI, DXD,DXR,CMS,CMK,MCS,HDS, SCS*	Wang et al., 2021
*D. officinale*	Illumina HiSeq 4,000	Precursor of terpenoid	*TPS-a, TPS-b, TPS-c, TPS-e/f*	Li et al., 2021

## Application of Synthetic Biology in the Large-Scale Production of *Dendrobium* alkaloids

Next generation sequencing technology provided a strong foundation for the acquisition of high-quality genome datasets of the medicinal plants, which facilitated the investigation of crucial genes in the secondary metabolism (Goossens and Rischer, 2007; Liu et al., 2020b). However, the industry still faces constraints, such as lack of raw materials and a long cultivated period. Meanwhile, alkaloid contents in most *Dendrobium* spp. are generally limited, which makes it tricky to obtain a large quantity of alkaloids rapidly. The utilization of microbial fermentation plants and synthetic biology can provide technological support for the secondary development and sustainable utilization of *Dendrobium* resources (Ehrenworth and Peralta-Yahya, 2017; Li et al., 2018a). Based on existing studies, there are two main technological methods for using synthetic biology in natural products: one is the heterologous expression of synthetic gene clusters of natural products in suitable chassis cells (Ro et al., 2006; Dangel et al., 2010; Gwak et al., 2017); the other is to introduce positive regulatory regulatory elements or delete negative regulatory elements in the original host to achieve regulation or overexpression of related genes (Zhuang et al., 2017; Ma et al., 2018). Since the synthetic route of natural products is generally extended, the problem of heavy metabolic load and low yield of single bacteria are often stressful to overcome. The exploration of multicellular systems provides flexibility and personalization for the production of such complex substances (Li et al., 2018b). By rationally designing and constructing an artificial multi-cell culture system, the metabolic pathways could be dispersed and assembled into multiple independent cells, which can lower the metabolic burden of a single bacteria. The optimal module combination could be achieved by engineering and maximizing the metabolic capacity of a single chassis cell (Zhang and Wang, 2016). The goal of synthetic biology has gradually evolved from the synthesis of biological components and devices (the first stage of synthetic biology) to the construction of multicellular life systems (the second stage of synthetic biology; Paddon et al., 2013; Ma et al., 2019b). With the gradual development of gene editing and *de novo* synthesis technologies, the chassis of synthetic biology is becoming more abundant, mainly including microbial chassis (*Saccharomyces cerevisiae* and *Escherichia coli*, etc.) and plant chassis (tobacco, suspension cells, hairy roots, etc.). The heterologous synthesis of iridoids, isoflavin, and vinblastine in yeast and the heterologous synthesis of triterpenoids, such as artemisinin and its precursors, β-amyrin and oleanolic acid in tobacco, provide scientific reference for *in vitro* synthesis of dendrobium alkaloids (Farhi et al., 2011; Miettinen et al., 2014; Brown et al., 2015; Fuentes et al., 2016; Malhotra et al., 2016; Kumar et al., 2018; Saiman et al., 2018).

Recent research found that specialized metabolic enzymes usually clustered in a few chromosomes, forming metabolic gene clusters. These gene clusters are widely present in the biosynthesis pathways of secondary metabolites in dicotyledonous and monocotyledonous plants (Nützmann et al., 2016). Generally, genes in some gene clusters are arranged continuously and tightly on chromosomes (Polturak and Osbourn, 2021). The thaliano-synthetic gene cluster in over 80% of *Arabidopsis* ecotypes, the acylated tyramine gene cluster in rice, and the diterpene casbene gene cluster present a continuous arrangement (Zhan et al., 2020; Shen et al., 2021). Some gene clusters are scattered in distant areas, such as the genes that synthesize the sterol alkaloid α-solanine in tomato clusters on chromosome 7 (Itkin et al., 2013). This encompasses two upstream pathway genes, *GAME11* and *GAME6*, as well as four glycosyltransferase genes, *GAME1*, *GAME17*, *GAME18*, and *GAME2*. The other two structural genes, *GAME12* and *GAME4*, which encode aminotransferases, are adjacent to each other on chromosome 12. Based on genome and transcriptome co-expression analysis, it is feasible to carry out initial mining of gene clusters of specific metabolic pathways (Jeon et al., 2020; Liu et al., 2020c). Some software and algorithms are capable of rapidly identifying potential gene clusters in non-model plants. Plant Cluster Finder is a more developed tool that has studied 18 plants and identified approximately 12,000 possible biosynthetic gene clusters (Schläpfer et al., 2017). PlantiSMASH and PhytoClust employ a more accurate hidden Markov model to identify distinct biosynthetic enzymes and integrate the gene locations to predict candidate gene clusters (Kautsar et al., 2017; Töpfer et al., 2017). It should be noted that *Dendrobium* alkaloids are mostly composed of TIAs and sesquiterpene alkaloids (Table 1). By altering particular critical enzymes in the MEP and MVA pathways, heterologous expression strategies can supply richer terpene precursors for the production of these two kinds of alkaloids (Zhu et al., 2014).

## Conclusion and Future Challenges

With the rapid development of sequencing technology and various “omics” technologies, plant-specific metabolic biosynthesis has rapidly progressed from small-scale sequencing and functional identification of individual genes to the large-scale sequencing and comparative genomics studies. Transcriptomics and metabolomics-associated analysis, including co-expression and co-response analysis, have been successfully employed in the functional identification of unknown genes in many non-model plants. The completion of high-quality genome sequencing and re-sequencing of natural populations has also provided considerable prospects for the exploration of crop metabolic pathways and valuable Chinese herbal medicines. The metabolome genome-wide association study (mGWAS) is a new approach to decipher in-depth analyses of complicated metabolic pathways and their regulatory mechanisms, as well as other basic theoretical studies. However, this technology is rarely applied in the research of active specific metabolites of medicinal plants. The main reason for this is that medicinal plants, unlike commercial crops, do not typically have systematic classification and whole germplasm lines. Synthetic biology is the design or modification of living systems at the molecular level to meet certain purposes. Plants can transform light energy and CO_2_ from the environment into their own energy and carbon sources. The use of synthetic biology to rebuild metabolic networks in plant cells and reroute carbon flow to more useful plant-specific metabolites has enormous potential. The convergence of cutting-edge technologies, such as improved genome editing, sophisticated gene module assembly, tobacco transient expression technology, rapid and efficient genetic modification with magnetic nanoparticles as carriers, and computer-aided design, will propel synthetic biology into the next era in the future.

## Author Contributions

CS, QJ, and YC discussed the writing plan. CS and JM drafted the manuscript. CS, JM, GL, HP, and YZ edited the manuscript. BH and QJ acquired the funding. All authors have read, reviewed, and approved the submitted version.

## Funding

This work was supported by China Agricultural Research System of MOF and MARA. The authors are also grateful for the financial support of National Industry Technology System of Traditional Chinese Medicine (CARS-21), Anhui University Collaborative Innovation Project (GXXT-2019-043 and GXXT-2019-049), High-level Talents Research Initiation Funding Project of West Anhui University (WGKQ2022025), and Natural Science Foundation of Anhui (2108085MC80).

## Conflict of Interest

The authors declare that the research was conducted in the absence of any commercial or financial relationships that could be construed as a potential conflict of interest.

## Publisher’s Note

All claims expressed in this article are solely those of the authors and do not necessarily represent those of their affiliated organizations, or those of the publisher, the editors and the reviewers. Any product that may be evaluated in this article, or claim that may be made by its manufacturer, is not guaranteed or endorsed by the publisher.
